# Water-specific toxicity factor and index for heavy metal risk assessment: application to urban lakes in Chennai, India

**DOI:** 10.1007/s10653-026-03298-2

**Published:** 2026-07-08

**Authors:** Daniel Rosado, Indumathi Nambi, Nicola Fohrer

**Affiliations:** 1 Department of Hydrology and Water Resources Management, Institute for Natural Resource Conservation, Kiel University, Olshausenstr. 75, 24118 Kiel, Germany; 2 Department of Civil Engineering, Indian Institute of Technology Madras, 600036 Chennai, India; 3 Department of Chemical and Environmental Engineering, Higher Technical School of Engineering, Universidad de Sevilla, Camino de los Descubrimientos S/N. 41092, Seville, Spain

**Keywords:** Heavy metal pollution, Urban lakes, Toxicity index, Pallikaranai catchment, Water quality, Sediment contamination

## Abstract

Heavy metals released into aquatic environments disrupt ecological balance. Urban lakes in the Pallikaranai catchment, Chennai, India, face pollution pressures from rapid urbanization. This study introduces a water-specific toxicity factor and toxicity index to assess toxicity risks posed by heavy metals in surface waters to aquatic life. Results revealed considerable variability in in situ water quality parameters, including pH, electrical conductivity, and dissolved oxygen. pH was mostly neutral to alkaline, with some lakes experiencing anoxia while others exceeded 10 mg/L oxygen. Pb and Cu posed the highest toxicity risks in water, with several samples reaching Class II and III in the United Nations Economic Commission for Europe (UNECE) classification, indicating potential toxicity risks. In sediments, Cr and Ni posed the highest toxicity risks, exceeding the PEC in all samples and Cu and Zn frequently exceeding sediment quality thresholds. Exceeding the TEC indicates possible toxic effects, while surpassing the PEC suggests probable toxic effects to aquatic biota. Compared to other polluted urban water bodies globally, the Pallikaranai catchment exhibited relatively low water contamination and high sediment contamination, particularly for Cr, Cu, Ni, and Zn. The newly developed water toxicity index, based on UNECE guidelines, indicated varying levels of potential ecological risk across lakes. This study highlights the need for monitoring and effective pollution mitigation strategies to protect the aquatic ecosystems of the Pallikaranai catchment. The proposed toxicity index provides a practical screening tool for assessing and prioritizing heavy metal risks in urban aquatic environments.

**Supplementary Information:**

The online version contains supplementary material available at 10.1007/s10653-026-03298-2.

## Introduction

Aquatic ecosystems worldwide face significant threats from heavy metal pollution, posing remarkable risks to ecosystem health and human well-being (Adeleye et al., 2024; Anetor et al., 2022; Piwowarska et al., 2024; Zhang et al., 2025). Heavy metals (in this article, the term heavy metal includes metals such as Al, As, Cr, Cu, Fe, Mn, Ni, Pb, and Zn, measured in this work, and metalloids, i.e. As, etc.) are contaminants in water bodies, originating from industrial discharges, urban runoff, mining, agriculture (such as the use of fertilizers and pesticides), atmospheric deposition (such as gases from vehicle emissions) and natural weathering of rocks and soils (Häder et al., 2020; Jaafar et al., 2023; Kapoor & Singh, 2021; Kumar et al., 2023; Rincon-Vasquez et al., 2025). Once introduced into the water of aquatic environments, these metals can accumulate in sediments, posing a persistent threat to ecosystem (Kong et al., 2023; Miranda et al., 2021). Moreover, their potential bioaccumulation through the food chain further amplifies the risks to aquatic biota, leading to adverse effects on reproduction, growth, and overall population dynamics (Roy, 2024; Sharma et al., 2024; Zaynab et al., 2022). Consequently, understanding and mitigating the impacts of heavy metal pollution on aquatic ecosystems are necessary for sustainable management and conservation efforts (Aziz et al., 2023; Rezanezhad et al., 2025).

Assessing heavy metal pollution in water is often conducted using indices that aggregate data in one or a few numerical values, facilitating communication and understanding, particularly for non-experts (Ahirvar et al., 2023; Zhang et al., 2023). Many indices focus on the anthropogenic increase of metal concentration and thus, evaluate enrichment relative to background levels. Examples include the Heavy Metal Pollution Index (HPI), which uses drinking water limits as background values, and the Heavy Metal Evaluation Index (HEI) and Degree of Contamination (Cd), both of which use the maximum admissible concentration as the baseline. However, indices should not only measure metal concentrations or enrichment levels but also assess and communicate the potential toxicity risk to aquatic biota (Birch, 2023).

In this context, many countries have established maximum allowable levels of heavy metals in their surface waters to protect aquatic biota. For instance, the European Union has set concentration limits for certain heavy metals in water under the Water Framework Directive (European Parliament, 2000) and its amendments (European Parliament, 2013). Similarly, the US Environmental Protection Agency (USEPA) has defined thresholds for acute and chronic toxicity for various heavy metals in their National Recommended Aquatic Life Criteria Table (USEPA, 2026a). However, frameworks specifically addressing the toxicity risk of heavy metal concentrations on aquatic species remain relatively scarce. One notable effort was undertaken by the United Nations Economic Commission for Europe, resulting in detailed water quality guidelines (UNECE, 1993). More recently, several studies have proposed local-scale toxicity risk thresholds for specific heavy metals (Lee et al., 2020; Li et al., 2021). Authorities and policymakers require indices that are straightforward to calculate and interpret, enabling them to effectively communicate pollution status and prioritize management actions to mitigate the ecological and human health risks associated with heavy metal pollution in aquatic environments (Ogwu et al., 2025; Saroop & Tamchos, 2021; Zhang et al., 2023).

Heavy metal pollution represents a significant environmental concern in India due to rapid industrialization and urbanization (Adimalla, 2020; Kumar et al., 2020). Indian urban areas like Chennai are particularly susceptible to heavy metal contamination because of a prompt industrial and urban development and a high population density (Adimalla, 2020; Asim & Nageswara Rao, 2021; Kumar et al., 2022; Mishra et al., 2022). Some studies have reported on the high levels of heavy metals in the water bodies of Chennai (Rosado et al., 2024b). In the Sembakkam lake, part of the Pallikaranai catchment, Pb in water reached levels that might bring chronic toxic effects. In sediments, Ni reached values that may imply a risk for chronic toxicity, and Cr and Cu had a probability of occasional chronic toxic exposure to the biota of the lake (Rosado et al., 2024a). In Chembarambakkam lake, where water is collected for drinking water supply, a study reported pre-monsoon and post-monsoon concentrations of Cu and Ni exceeding the World Health Organization thresholds for drinking water, with Fe levels also surpassing these limits in the pre-monsoon season (Kiran & Sivakumar, 2023). Additionally, high levels of Cu and Pb have been reported in lakes of south Chennai (Lakshmi et al., 2018).

The Pallikaranai catchment, situated within the city of Chennai, includes a network of interconnected urban lakes, locally known as tanks, and the Pallikaranai Marshland, which was designated a Ramsar site in July 2022, that play a vital role in biodiversity conservation in general and as a crucial stopover point for migratory birds in particular (Jessieleena & Nambi, 2023; Rosado et al., 2024a). However, increasing anthropogenic pressures, including heavy metal pollution by untreated sewage discharge, waste dumping and others, threaten these ecosystems, highlighting the urgent need for comprehensive research and conservation efforts to safeguard Chennai's natural heritage (Sun et al., 2023).

The coexistence of urban lakes and residential areas in the city increases the potential exposure of humans and wildlife to heavy metal and other pollutants, underscoring the urgent need for robust monitoring and management strategies (Chandrasekar et al., 2020; Prapanchan et al., 2023; Sun et al., 2023).

While toxicity-based approaches have been increasingly applied to sediments, comparable tools for integrating and communicating heavy metal toxicity risks in surface waters remain less developed. Therefore, a water-specific toxicity factor and toxicity index can complement sediment-based assessments and provide a more complete picture of metal-related ecological risks in aquatic ecosystems.

The aim of this study is to develop a water-specific toxicity factor and toxicity index to assess heavy metal risks to aquatic biota in surface waters and to apply them to the lakes of the Pallikaranai catchment, an area with remarkable ecological value that is threatened by rapid urbanization while scientific literature remains scarce.

## Materials and methods

### Study area

The Indian city of Chennai is located on the southeastern coast of India (13°05′ N; 80°18′ E), on a flat area with an average elevation of 6.7 m.a.s.l. With a population of 7 million in the Chennai district and 8.7 million in the metropolitan area, Chennai is the fourth largest city in India, behind Delhi, Mumbai, and Kolkata (Directorate of Census Operations Tamil Nadu, 2011). The city experiences a tropical wet and dry climate (Köppen: Aw) characterized by stable temperatures throughout the year and a monsoon season (Rajanikanth & Rajini Kanth, 2020).

**Fig. 1:**
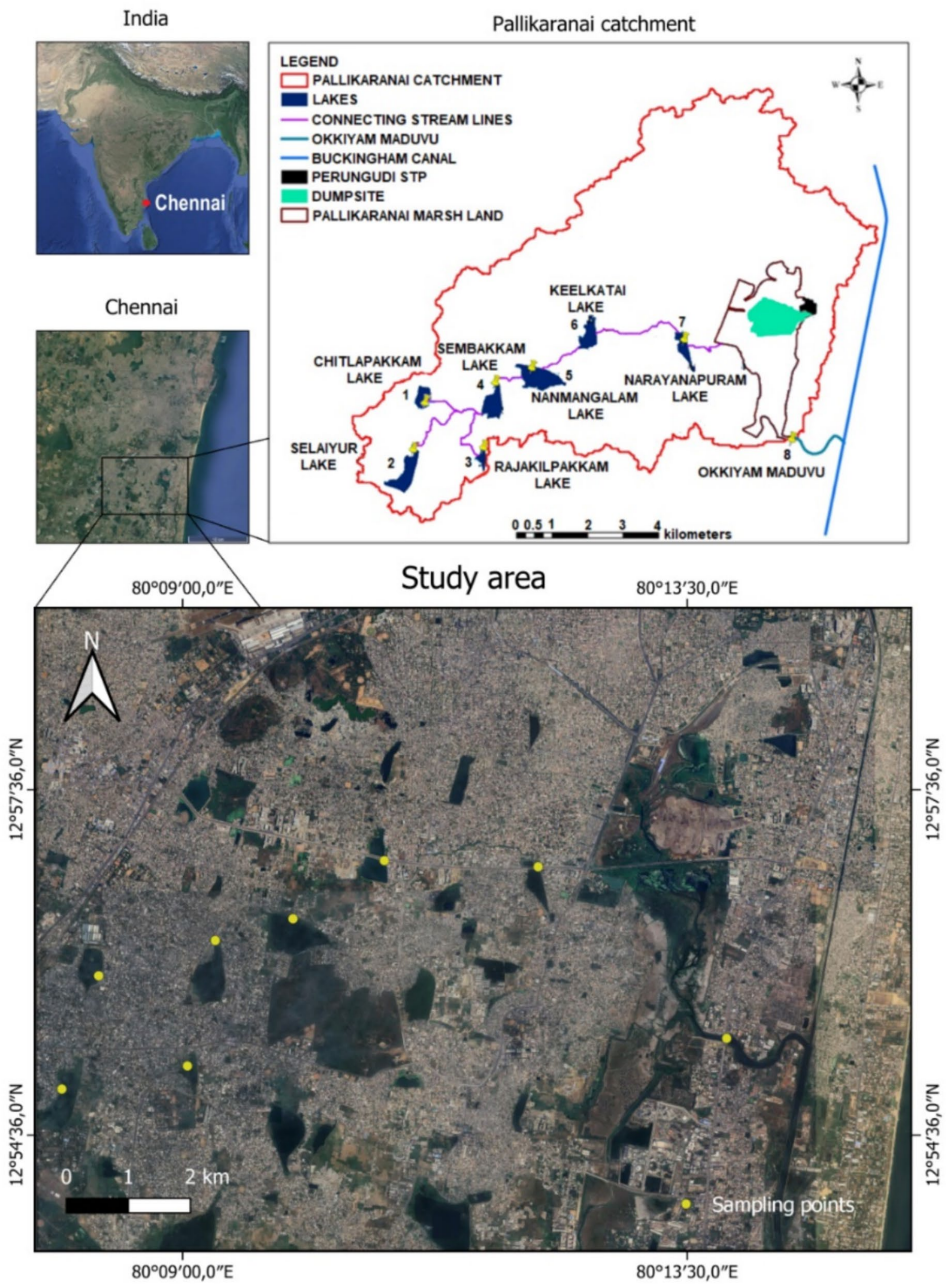
Sampling points in the lakes of the Pallikaranai Catchment, Chennai, India

The Pallikaranai catchment (Fig. 1), located within the city, contains seven interconnected lakes as a cascade system that ultimately drain into the Pallikaranai marshland and then into the Bay of Bengal via the Buckingham Canal and Adyar River (Jessieleena & Nambi, 2023; Shekhar, 2020; Tamil Nadu State Wetland Authority, 2024). The upstream lakes—Chitlapakkam, Selaiyur, and Rajakilpakkam—discharge their waters into Sembakkam Lake. The outflow from Sembakkam Lake goes to Nanmangalam Lake, subsequently to Keelkatalai, Narayanapuram, and finally to Okkiyam Maduvu, which is the outlet of the catchment and the connection with the Pallikaranai marshland (Sun et al., 2023).

Historically, the catchment was an agricultural area where these artificial lakes were constructed to store monsoon water to irrigate fields during the dry season (Palanisami & Nagothu, 2024; Rosado et al., 2024b). However, due to rapid and unplanned urbanization, many lakes have lost their original function and have become receiving environments of untreated sewage and waste from nearby residents, resulting in severe pollution, as observed in Sembakkam Lake, Rajakilpakkam, Madipakkam, and Medavakkam (Palanisami & Nagothu, 2024; Rosado et al., 2024a).

### In situ parameters, sampling and sample pre-treatment

Eight sampling points were selected throughout the Pallikaranai catchment, seven from urban lakes and one from Okkiyam Maduvu, the outlet of the catchment and its connection to the Pallikaranai marshland. The coordinates of these locations are depicted in Fig. 1 and detailed in the supplementary material (Table S1). Sampling points in lakes were located either directly at the lake outlets or from adjacent points when the outlets were inaccessible. Two sampling campaigns were conducted, on October 2, 2022, and on November 3, 2022. At each sampling point, in-situ water quality parameters were measured, and samples of water and sediment were collected. Immediately after collection, each sample was placed in a dark cooler at 4 °C to prevent degradation. Samples were transported to the laboratories of the Civil Engineering Department at the Indian Institute of Technology Madras, ensuring they arrived no later than three hours post-collection.

In-situ water quality parameters, specifically pH, electrical conductivity, dissolved oxygen, and temperature were measured using a WTW 3630 IDS portable multiparameter meter (Xylem Analytics, Germany) at each designated sampling point. Water samples were collected in 50 ml polyethylene bottles, which had been pre-cleaned in the laboratory with 10% HNO_3_ followed by Milli-Q water and then rinsed three times with lake water at the sampling point. Once collected, samples were transported to the laboratory, where they were filtered through 0.45 μm pore Teflon filters, acidified to a pH of 2 or lower using HNO_3_, and stored at 4 °C until analysis. These procedures align with ISO standard 5667 parts 1, 3, and 4, which represent best practices in environmental sample handling (Noble et al., 2020; Romero-Mujalli et al., 2022; Rosado et al., 2024a).

Sediment samples were collected using a Van Veen grab, ensuring that only samples with adequate penetration were retained. Each sample was obtained by aggregating, mixing, and homogenizing five full grabs on-site to achieve a representative sample from each point. The samples were stored in a dark cooler at 4 °C and transported to the laboratory. There, the sediment samples were dried at 60 °C, disaggregated with an agate mortar, and sieved to obtain the < 63 μm fraction. Metal concentrations were calculated based on dry weight sediment (105 °C). These procedures comply with ISO standards 5667:2023 parts 1, 12, and 15, and to common practices in existing scientific literature (Felizardo et al., 2021; Rincon-Vasquez et al., 2025; Rosado et al., 2024a).

### Sediment digestions

For the digestion process, 0.2 g of each dried and sieved sediment sample underwent a controlled digestion in a PicoTrace® digestion block. Each sample was treated in a closed Teflon vessel with 5 ml of 65% Suprapur nitric acid (Merck, Germany) and maintained at a temperature of 140 °C for 16 h. After the digestion period, the apparatus was allowed to cool to room temperature. The resulting digestates were then filtered and made up to 50 ml in volumetric flasks with Milli-Q water, after which they were transferred to polypropylene bottles and refrigerated at 4 °C until metal analysis. To ensure analytical accuracy, each digestion batch included a blank vessel containing only 5 ml of 65% Suprapur nitric acid, as well as a reference material vessel containing SO-4 reference soil from the Canadian Certified Reference Materials Project. The recovery rates for each metal from the reference material consistently ranged between 90–110% (Table S2), confirming the reliability of the digestion procedure (Barquero et al., 2025; Shahbazi & Beheshti, 2019).

### Heavy metals measurements

The concentrations of Al, As, Cr, Cu, Fe, Mn, Ni, Pb, and Zn in both water samples and sediment digestates were quantified using a Thermo Scientific® ICP-OES iCAP 6000 Series, equipped with both axial and radial views. The instrument was calibrated using Certipur® ICP multi-element standard solutions IV and XIII from Merck (Dashtizadeh et al., 2019).

### Data analysis

Descriptive statistics, including average, median, standard deviation, minimum, maximum and coefficient of variation, were calculated for the in situ parameters and heavy metal concentrations in water and sediments, both separately for each sampling campaign and for the complete dataset. Differences between sampling campaigns were assessed using paired tests because the same sampling points were monitored in both campaigns. Normality of paired differences was evaluated using the Shapiro–Wilk test. When paired differences were normally distributed, paired t-tests were applied; otherwise, Wilcoxon signed-rank tests were used. Benjamini–Hochberg adjusted p-values were calculated to account for multiple comparisons. Paired comparisons between the two sampling campaigns did not reveal significant differences after adjustment for multiple comparisons. Therefore, average values from both campaigns were used for the calculation of the toxicity factors and toxicity indices.

#### Comparison to guidelines

In order to evaluate the toxicity risk, heavy metal concentrations in water were compared with the United Nations Economic Commission for Europe (UNECE, 1993) guidelines for the classification of surface freshwater quality for the maintenance of aquatic life. These UNECE guidelines classify surface freshwater quality into five classes according to the potential toxicity risk posed by heavy metal concentrations to aquatic species, thereby providing a structured framework for evaluating ecological threats in aquatic ecosystems (Bearcock et al., 2017; Dong et al., 2020; Elmorsi et al., 2019; Fetoshi et al., 2024; Malsiu et al., 2020; Shcherbakov et al., 2025; Shuhaimi-Othman et al., 2012).

For sediments, heavy metal concentrations were evaluated against the consensus-based sediment quality guidelines developed by MacDonald et al. (2000). These guidelines define two critical thresholds: the Threshold Effect Concentration (TEC) and the Probable Effect Concentration (PEC). The TEC refers to the concentration below which adverse effects are unlikely to occur. TEC values, in mg/kg, are: As (9.79), Cd (0.99), Cr (43.4), Cu (31.6), Pb (35.8), Ni (22.7), Zn (121). Conversely, the PEC is the concentration above which adverse effects are expected to occur more often than not. PEC values, in mg/kg, are: As (33), Cd (4.98), Cr (111), Cu (149), Pb (128), Ni (48.6), Zn (459) (MacDonald et al., 2000). Therefore, in the interval in the middle toxic effects are possible but not always present (Long & MacDonald, 1998).

#### Toxicity factor and toxicity index for heavy metals in water

In this study, the toxicity factor (Tf) and toxicity index (TI) for heavy metals in surface waters were developed using the water toxicity guidelines established by the UNECE (1993). This approach follows the same rationale as sediment toxicity indices based on threshold values, but adapts the normalization procedure to the class-based structure of the UNECE water-quality framework. The toxicity factor normalizes the metal content in water according to these guidelines and is calculated as follows:

$$\begin{aligned}&\text{If } C_{m,s} < T_{m,\mathrm{class}=2}: \\ &Tf_{m,s} = 1 + \left( \frac{C_{m,s}}{T_{m,\mathrm{class}=2}} \right) \\ &\text{and } 1 < Tf_{m,s} < 2\end{aligned}$$

$$\begin{aligned}&\text{If } T_{m,\mathrm{class}=2} \le C_{m,s} < T_{m,\mathrm{class}=5}: \\ &Tf_{m,s} = \mathrm{class} + \left( \frac{C_{m,s} - T_{m,\mathrm{class}}}{T_{m,\mathrm{class}+1} - T_{m,\mathrm{class}}} \right) \\ &\text{and } \mathrm{class} \le Tf_{m,s} < \mathrm{class} + 1\end{aligned}$$

$$\begin{aligned}&\text{If } C_{m,s} \ge T_{m,\mathrm{class}=5}: \\ &Tf_{m,s} = 5 + \left( \frac{C_{m,s} - T_{m,\mathrm{class}}}{T_{m,\mathrm{class}=5}} \right) \\ &\text{and } Tf_{m,s} \ge 5\end{aligned}$$

where:

- ${C}_{m,s}$ is the concentration of the metal *m* in sample *s*.
- class is the *class* that the metal concentration belongs to, according to the guidelines developed by (UNECE, 1993) and shown in Table 1.
- ${T}_{m,class}$ is the lower threshold of metal *m* for the respective *class*, according to the guidelines developed by (UNECE, 1993) and displayed in Table 1.
- ${Tf}_{m,s}$ is the toxicity factor of the metal *m* in the sample *s*.

**Table 1:** Heavy metal freshwater quality standards (μg/L) of the United Nations Economic Commission for Europe for the maintenance of aquatic life (UNECE, 1993)

Element	Class I	Class II	Class III	Class IV	Class V
As	< 10	10—100	100—190	190—360	> 360
Cd	< 0.07	0.07—0.53	0.53—1.1	1.1—3.9	> 3.9
Cr	< 1	1.0—6	6.0—11	11.0—16	> 16
Cu	< 2	2.0—7	7.0—12	12.0—18	> 18
Pb	< 0.1	0.1—1.6	1.6—3.2	3.2—82	> 82
Hg	< 0.003	0.003—0.007	0.007—0.012	0.012—2.4	> 2.4
Ni	< 15	15—87	87—160	160—1400	> 1400
Zn	< 45	45—77	77—110	110—120	> 120

Class I: No anthropogenic pollution with inorganic matter; Class II: Concentrations are below the midpoint between natural and chronically toxic levels; Class III: Concentrations are above the midpoint between natural and chronically toxic levels; Class IV: Excursions beyond chronic criteria occur, but do not establish chronically toxic conditions in terms of concentration levels, duration or frequency; Class V: Excursions beyond chronic criteria concentrations allow acutely toxic conditions in terms of concentration levels, duration or frequency

The toxicity factors are interpreted according to the class system provided in the UNECE guidelines.

To express the combined toxicity of several metals in a single figure, the toxicity factors of one water sample were integrated into the toxicity index (TI). This index is the geometric mean of the toxicity factors for a sample:

$${Ti}_{s}={({Tf}_{m1,s}\times {Tf}_{m2,s}\times \dots \times {Tf}_{mi,s} )}^{1/i}$$

where:.

- ${Ti}_{s}$ is the toxicity index in the sample *s*,
- Tf_m1,s_ is the toxicity factor of the metal *m1* in the sample *s*,
- Tf_mi,s_ is the toxicity factor of the i-th metal *mi* in the sample *s*.

The toxicity index values are categorized in the same manner as the toxicity factors into five categories, facilitating the understanding of water toxicity risks.

Paired comparisons between the two sampling campaigns did not reveal significant differences in water metal concentrations after adjustment for multiple comparisons. Therefore, the toxicity factor and toxicity index for water were calculated using the average metal concentrations from both campaigns.

#### Toxicity factor and toxicity index for heavy metals in sediment

The toxicity factor and toxicity index for assessing toxicity risks from heavy metals in sediments were calculated following Rosado et al. (2026).

The toxicity factor normalizes the metal content in sediments with the TEC and PEC for each metal developed by MacDonald et al. (2000) using the following equations:

$$\begin{aligned}&\text{If } C_{m,s} < TEC_{m}: \\ &Tf_{m,s} = \frac{C_{m,s}}{TEC_{m}} \\ &\text{and } 0 < Tf_{m,s} < 1\end{aligned}$$

$$\begin{aligned}&\text{If } TEC_{m} \le C_{m,s} < PEC_{m}: \\ &Tf_{m,s} = 1 + \left( \frac{C_{m,s} - TEC_{m}}{PEC_{m} - TEC_{m}} \right) \\ &\text{and } 1 \le Tf_{m,s} < 2\end{aligned}$$

$$\begin{aligned}&\text{If } C_{m,s} \ge PEC_{m}: \\ &Tf_{m,s} = 2 + \left( \frac{C_{m,s} - TEC_{m}}{PEC_{m}} \right) \\ &\text{and } Tf_{m,s} \ge 2\end{aligned}$$

where:

- $C_{m,s}$ is the concentration of the metal *m* in the sample *s*.
- $TEC_{m}$ is the TEC of the metal *m*.
- $PEC_{m}$ is the PEC of the metal *m*.
- $Tf_{m,s}$ is the toxicity factor of the metal *m* in the sample *s*.

The toxicity factors are interpreted as follows: no sediment toxicity (Tf < 1), possible sediment toxicity (1 ≤ Tf < 2) and probable sediment toxicity (Tf ≥ 2) according to the interpretation of Rosado et al. (2026).

To express the combined toxicity of several metals in a single figure, the toxicity factors of one sediment sample were integrated into the toxicity index. This index is the geometric mean of the toxicity factors for a sample:

$$Ti_{s} = \left( {Tf_{m1,s} \times Tf_{m2,s} \times \ldots \times Tf_{mi,s} } \right)^{1/i}$$

where.

- $Ti_{s}$ is the toxicity index in the sample *s*,
- Tf_m1,s_ is the toxicity factor of the metal *m1* in the sample *s*,
- and Tf_mi,s_ is the toxicity factor of the i-th metal *mi* in the sample *s*.

The toxicity index values are categorized in the same manner as the toxicity factors into three categories, facilitating the understanding of sediment toxicity risks.

Paired comparisons between the two sampling campaigns did not reveal significant differences in sediment metal concentrations after adjustment for multiple comparisons. Therefore, the sediment toxicity factor and toxicity index were calculated using the average metal concentrations from both campaigns.

RStudio software version 2023.06.1 + 524 (R Development Core Team, 2023) was used to produce the graphs and QGIS 3.28 to produce the maps.

## Results and discussion

### In situ parameters

In situ parameters in the lakes of the Pallikaranai catchment exhibited considerable variability within lakes but remained relatively stable across both sampling campaigns, as shown in the supplementary material (Table S3). Regarding pH, the average across the catchment was 8.07 in the first sampling campaign and 8.06 in the second, indicating neutral-alkaline conditions. Selaiyur and Rajakilpakkam lakes, located in the upper part of the catchment, as well as Okkiyam maduvu, showed the lowest pH values, ranging between 7 and 7.6 in both samplings. From these points downstream, pH values tended to increase. Middle lakes displayed shifts in pH between samplings, with Sembakkam dropping from 8.24 to 7.62 and Nanmangalam rising from 7.91 to 8.59. Downstream lakes, Keelkatai and Narayanapuram, had the highest pH values, at 8.8 and 9.05, respectively. Chitlapakkam Lake, despite being upstream, presented high pH levels (8.55 and 8.57), similar to those found in the downstream lakes.

Only the pH measurement in Narayanapuram lake during the first sampling campaign (9.02) slightly exceeded the range (6.5–9) defined by the USEPA as suitable for aquatic life in the National Recommended Aquatic Life Criteria Table (USEPA, 2026a). Considering the more restrictive range (6.5–8.5) defined by the Indian Central Pollution Control Board (CPCB) for the propagation of wildlife and fisheries (BIS, 1982; CPCB, 1979), Chitlapakkam and the downstream lakes, Keelkatai and Narayanapuram lakes, failed to comply. Compared to other urban lakes in Chennai, the lakes in the Pallikaranai catchment showed less extreme values than the Velachery lake (8.87) and Perungudi (6.0), Karapakkam (6.1), Porur (6.2), Puzhal (7.0) and Nandhivaram (7.02) (Raji & Abraham, 2018).

Average electrical conductivity values (EC) were similar in both sampling campaigns, with 1344 µS/cm in the first and 1288 µS/cm in the second. A similar behavior as observed with pH was noted. Two upstream lakes, namely Selaiyur (1602 µS/cm) and Rajakilpakkam (1356 µS/cm), and Okkiyam maduvu displayed the highest average EC value (2335 µS/cm). In the middle lakes, Sembakkam also had high EC values (1435 µS/cm). Downstream, there was a decreasing trend in EC values, with Nanmangalam lake (1349 µS/cm) and the downstream lakes, Keelkatai (1000 µS/cm) and Narayanapuram lake (920 µS/cm) showing lower values. As it happened with pH, Chitlapakkam Lake, though upstream, had the lowest average EC and similar to downstream lakes: 673 µS/cm.

Only the EC in Okkiyam maduvu in both samplings, Selaiyur lake in first sampling and Sembakkam lake in second sampling exceeded the range defined by the USEPA (50–1500 µS/cm) for common EC in rivers of the United States (USEPA, 2026b). However, only Chitlapakkam and Narayanapuram lakes, those with the lowest ECs, complied with the more restrictive CPCB threshold value (1000 µS/cm) for the propagation of wildlife and fisheries (BIS, 1982; CPCB, 1979). Finally, none of the studied water bodies complied with the USEPA range (150–500 µS/cm) defined for US rivers with good mixed fisheries (USEPA, 2026b).

Temperatures were similar in different lakes in the first sampling (average 30.5 °C) and in the second (average 32.7 °C). Dissolved oxygen concentration averages were similar in both sampling campaigns, with 5.50 mg/L in the first and 5.16 mg/L in the second. Similar to the patterns observed with pH and EC, the upstream lakes, Selaiyur and Rajakilpakkam, along with Okkiyam Maduvu, exhibited the lowest dissolved oxygen concentrations, recorded at 0.24 mg/L, 0.22 mg/L, and 1.19 mg/L, respectively. There was an increasing trend in dissolved oxygen levels from upstream to downstream lakes. Middle lakes displayed intermediate oxygen levels, with Sembakkam at 6.30 mg/L and Nanmangalam at 6.34 mg/L. Downstream lakes, Keelkatai and Narayanapuram, had the highest oxygen values and saturations, at 10.50 mg/L (144.3%) and 10.54 mg/L (142.2%), respectively. Chitlapakkam Lake also showed an intermediate oxygen value of 7.31 mg/L. Oxygen values in Selaiyur and Rajakilpakkam lakes are considered devoid of life (< 1 mg/L) and of concern (1–3 mg/L) in Okkiyam Maduvu according to USEPA (2026c). Conversely, Keelkatai and Narayanapuram lakes are well above common oxygen scarcity thresholds. Chitlapakkam, Sembakkam and Nanmangalam lakes comply with the CPCB limits for the propagation of wildlife and fisheries (BIS, 1982; CPCB, 1979) and with the obligatory thresholds for fishes of the Cyprinidae family (4 mg/L) and Salmonids (6 mg/L) and should be able to sustain most forms of life requiring oxygen (European Council, 2006).

The lakes in the Pallikaranai catchment receive a remarkable amount of untreated sewage that adversely affects their water quality (Rahman et al., 2021; Rosado et al., 2024a). During the on-site inspection of the lakes, it was noted that downstream lakes with high oxygen concentrations displayed an intense green color, typically associated with algal blooms and eutrophication. This phenomenon can be linked to elevated nitrogen and phosphorus levels due to abundant sewage discharge in the catchment area (Mishra et al., 2023). Additionally, the higher pH values observed in these lakes may also be associated with eutrophication, as intense phytoplankton photosynthesis can consume dissolved CO₂, shift the carbonate equilibrium, and consequently increase water pH (Sakaguchi et al., 2023; Verspagen et al., 2014). However, in the lakes upstream, the sewage discharges and the subsequent input of nitrogen and phosphorus seem to drop the oxygen due to organic matter oxidation by heterotrophic bacteria (Zhang et al., 2020).

Descriptive statistics confirmed the high variability of several in situ parameters, particularly dissolved oxygen and oxygen saturation, which showed overall coefficient of variation values of 80.04% and 79.88%, respectively (Table S3).

### Water samples

#### Heavy metal concentrations in water

**Fig. 2:**
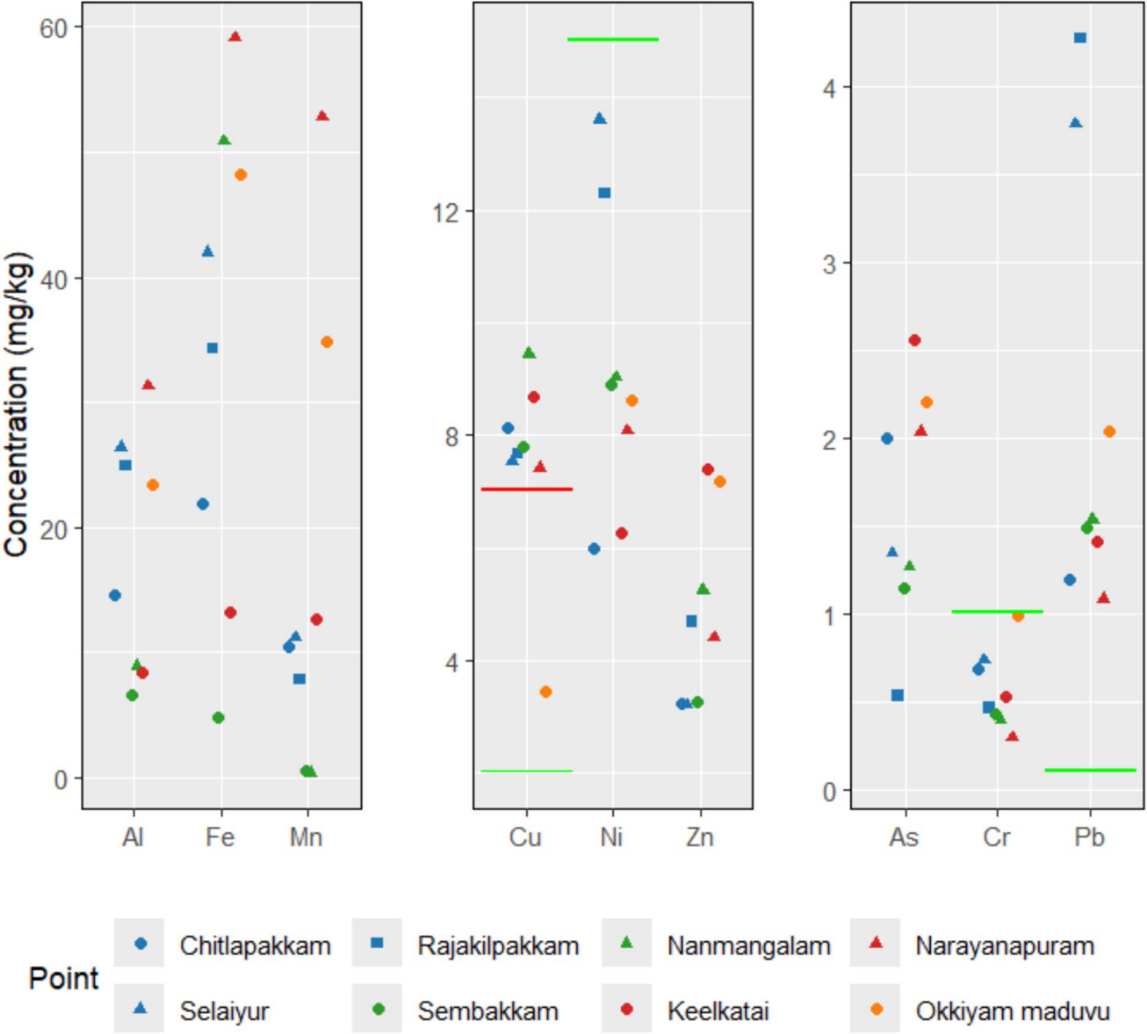
Heavy metal concentrations in the waters of the Pallikaranai catchment lakes, Chennai, India (dots), along with the thresholds proposed by the UNECE guidelines for the maintenance of aquatic life (UNECE, 1993) (lines). Blue dots represent upstream lakes, green dots represent the middle lakes, red dots represent downstream lakes, and orange dots represent the outlet of the catchment. Green lines indicate the Class 1–2 thresholds, and red lines indicate the Class 2–3 thresholds. Al, Fe, and Mn lack UNECE values. Missing threshold lines of Ni, Zn, As, Cr, and Pb indicate guideline values outside the displayed y-axis range

The water of the lakes of the Pallikaranai catchment presented the following average concentrations of heavy metals: Fe (34.3 µg/L) > Al (18.1 µg/L) > Mn (16.3 µg/L) > Ni (9.09 µg/L) > Cu (7.51 µg/L) > Zn (4.82 µg/L) > Pb (2.11 µg/L) > As (1.64 µg/L) > Cr (0.57 µg/L). These values are consistent with those reported in a previous study on Sembakkam Lake (Rosado et al., 2024a). Detailed data can be found in Fig. 2 and in the supplementary material (Table S4).

Ranges of heavy metal concentrations displayed considerable variability (Table 2). Descriptive statistics also indicated considerable spatial and temporal variability, especially for Mn, Fe, Al and Pb, which showed overall coefficient of variation values of 169.7%, 98.1%, 80.6% and 77.2%, respectively (Table S4).

**Table 2:** Heavy metal concentrations in water (μg/l) in urban lakes around the world

S. no	Study area	Al	Fe	Mn	As	Cr	Cu	Ni	Pb	Zn	References
1	Lakes Pallikaranai catchment (India)	5.8—48.9	1.1—101	0.2—96.7	0.2—2.9	0.1—1.5	1.7—12.5	4.6—16.4	0.6—6.3	1.7—13.0	Present study
2	Sembakkam Lake, Chennai India		2.2—95	0—17.3	0.1—2.7	0—1.7	1.5—6.3	4.1—8.7	0.3—5.4	1.6—14.6	Rosado et al. (2024a)
3	Wuhan urban lakes, China					1.60–4.16	0.65–4.91	0.81–13.9	0.08–7.00	6.96—10.3	Dou et al. (2022)
4	Seoul urban lakes, South Korea		2200–16000	160–950		4.4–29	13–70	2.3–17	6.8–25	19–130	Yang et al. (2016)
5	Korotoa urban river, Bogra, Bangladesh				10–92	33–126	23–119	9.3–71	8–64		Islam et al. (2015)
6	Twin lakes, Bhopal, India					30—80	20—70	80—350	50—280		Gupta et al. (2013)

When compared to other polluted urban water bodies globally, the heavy metal levels in the lakes of the Pallikaranai catchment are generally lower. For instance, in Twin Lakes, Bhopal, India, the concentrations of Cr, Cu, Ni, Pb are significantly higher, indicating additional pollution sources (Gupta et al., 2013). A similar situation is observed in the Korotoa River, Bogra, Bangladesh, where levels of As, Cr, Cu, Ni and Pb exceed those found in this study (Islam et al., 2015). In the urban lakes of Seoul, South Korea, heavy metal concentrations, except for Ni, are much higher compared to the Pallikaranai catchment (Yang et al., 2016). However, in the urban lakes of Wuhan, China, Ni and Pb concentrations are comparable to those in the Pallikaranai lakes (Dou et al., 2022). Although concentrations in water are lower than in other urban polluted lakes, the water in the lakes of the Pallikaranai catchment is affected by heavy metal pollution, necessitating ongoing monitoring and pollution mitigation strategies.

#### Comparison with water-quality guidelines

According to the heavy metal freshwater quality guidelines proposed by the United Nations Economic Commission for Europe (UNECE) for the maintenance of aquatic life (Table 1), most samples from the Pallikaranai catchment are unlikely to produce toxic effects on aquatic species. Specifically, the levels of As, Cr, Ni, and Zn fall within Class I, indicating an absence of toxic effects according to the UNECE framework. In the first sampling campaign, Cr levels in Okkiyam Maduvu slightly exceeded the Class I threshold and were categorized as Class II. Similarly, Ni levels in Selaiyur Lake during the first sampling and in Okkiyam Maduvu during the second sampling also fell into Class II, indicating levels below the midpoint between natural and chronically toxic levels. The situation is different for Pb and Cu. For Cu, most samples in the first campaign fell within Class II, while in the second campaign, most samples were within Class III, considered over the midpoint between natural and chronically toxic levels. In Nanmangalam Lake, Cu levels even reached Class IV, indicating excursions beyond chronic criteria. For Pb, concentrations in the first sampling campaign were categorized as Class II in three lakes, Class III in two, and Class IV in three. In the second sampling, Pb levels were categorized as Class II and III, with an equal distribution between these two classes.

Excess of metals in aquatic ecosystems can lead to bioaccumulation, beginning with benthic organisms that consume particulate matter and progressively increasing up the food chain (Qin & Tao, 2022). Although Cu is an essential element for living beings (Kumar et al., 2021; Shabbir et al., 2020), high levels of exposure can adversely affect plant growth and development, and in humans, it is associated with Wilson disease (Jomova et al., 2022). Pb can produce harmful effects on blood and kidney of animals as well as on their reproductive, nervous, and immune systems (Gudadhe et al., 2024). In humans, excessive Pb exposure is linked to neurological disorders and other health issues (Collin et al., 2022).

The high input of untreated sewage from nearby households has a remarkable impact on the water quality of the lakes of the Pallikaranai catchment as well as the waste that is frequently dumped along their banks (Sun et al., 2023), which are common sources of Cu in urban lakes (Li et al., 2020; Wang et al., 2022). Regarding Pb, there are several additional sources, such as leaching from pipes made of Pb or materials containing Pb, like Polyvinyl chloride (PVC), residual pollution from leaded gasoline, fly-ash deposition from coal-based power plants and erosion of soil particles containing Pb (Boyle et al., 2020; Nawrot et al., 2020; WHO, 2019; Wu et al., 2022).

#### Toxicity factor and toxicity index for water

**Fig. 3:**
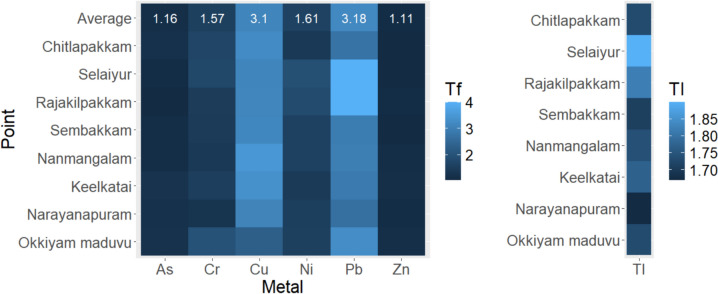
Heatmaps of the toxicity factors (Tfs) and toxicity index (TI) of heavy metals in water of the lakes of the Pallikaranai Catchment, Chennai, India as proposed in this article

The toxicity index values of metal concentrations in water (Fig. 3) revealed small differences among lakes that mostly align to Class I of the UNECE standards (Table S5). The upstream lakes, Selaiyur and Rajakilpakkam, had the highest average (1.88), excluding Chitlapakkam. This index value decreased in the middle section of the catchment, encompassing Sembakkam and Nanmangalam (1.73), as well as the downstream lakes, Keelkatai and Narayanapuram (1.72). However, the values increased again in Okkiyam Maduvu, reaching levels closer to the upstream lakes (1.79). Chitlapakkam Lake, despite being upstream, exhibited lower values similar to those found in the middle and downstream lakes (1.73). This trend mirrors the pattern observed for the in-situ parameters.

Although this study provides a useful framework for assessing heavy metal toxicity risks in urban lakes, the monitoring was based on two sampling campaigns and eight sampling points; therefore, seasonal variability and long-term temporal trends could not be fully assessed.

### Sediment samples

#### Heavy metal concentrations in sediments

**Fig. 4:**
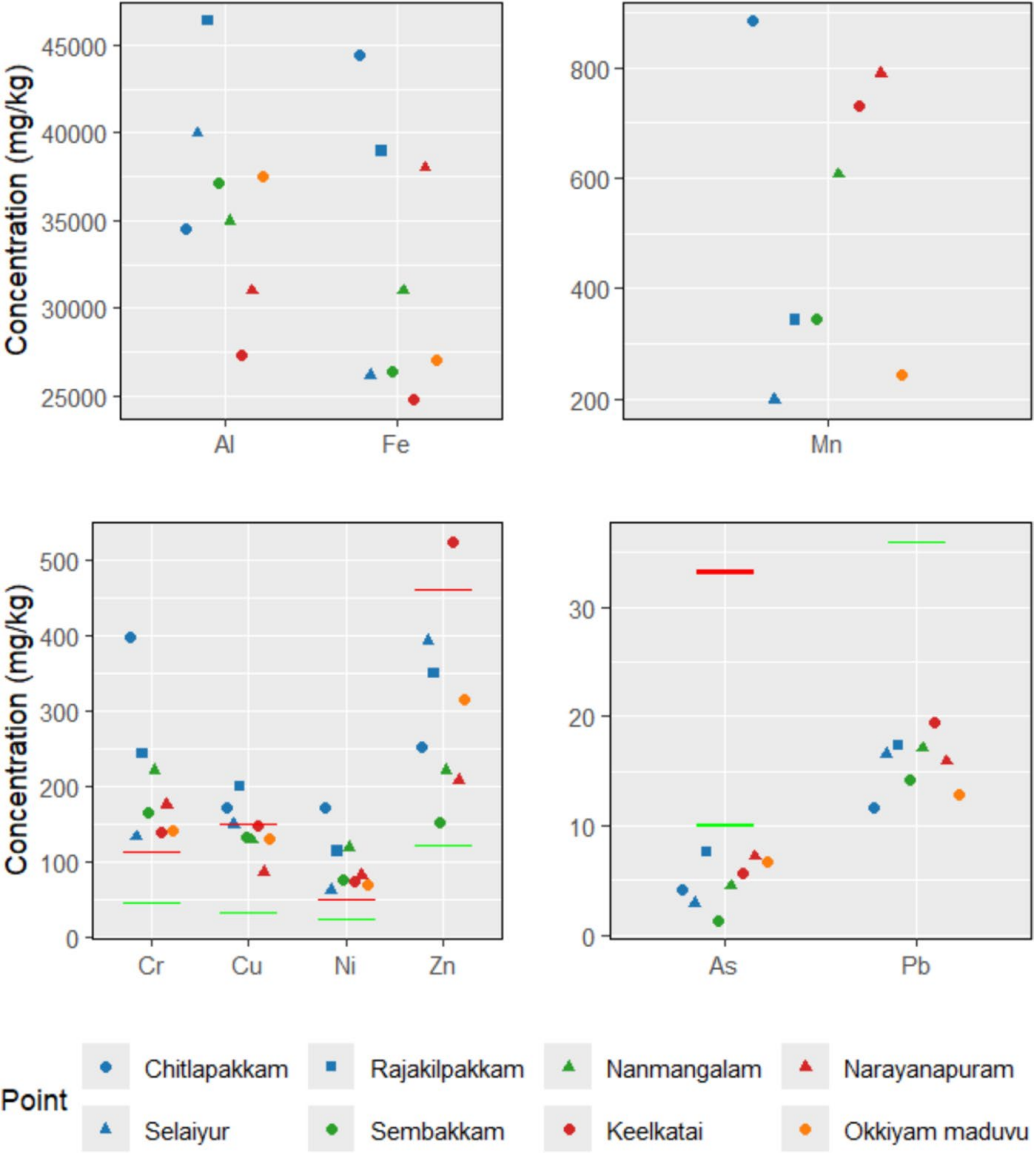
Heavy metal concentrations in sediments of the lakes of the Pallikaranai catchment, Chennai, India (dots), and Threshold Effect concentration (TEC, green lines) and Probable Effect Concentration (PEC, red lines) according to MacDonald et al. (2000). Blue dots represent upstream lakes, green dots represent middle lakes, red dots represent downstream lakes, and orange dots represent the outlet of the catchment. Al, Fe and Mn lack TEC and PEC values. The missing PEC line for Pb indicates a too high concentration that fall outside the range of the graph

The average concentrations of heavy metals in the sediments of the lakes of the Pallikaranai catchment were Al (36,098 mg/kg) > Fe (32,095 mg/kg) > Mn (517.6 mg/kg) > Zn (302.4 mg/kg) > Cr (202.5 mg/kg) > Cu (144.2 mg/kg) > Ni (99.65 mg/kg) > Pb (15.65 mg/kg) > As (5.01 mg/kg). These values are compatible with a previous study conducted in the Sembakkam lake (Rosado et al., 2024a). More data collected on heavy metal levels in the sediments can be found in Fig. 4 and in the supplementary material (Table S6).

As well as in waters, the heavy metal concentrations in the sediments of the urban lakes of the Pallikaranai catchment show considerable variability (Table 3) and aligns with findings from Sembakkam Lake (Rosado et al., 2024a). The highest overall variability was observed for Zn, Mn and As, with coefficient of variation values of 69%, 59% and 55.48%, respectively, indicating heterogeneous accumulation patterns among lakes (Table S6).

**Table 3:** Heavy metal concentrations in sediment (mg/kg) in urban lakes around the world

S. no	Study area	Al	Fe	Mn	As	Cr	Cu	Ni	Pb	Zn	References
1	Lakes Pallikaranai catchment, Chennai, India	26,546—57,687	22,298—52,029	151—1072	1.2—11.0	121–424	80—218	57—174	11—26	123—924	Present study
2	Seoul urban lakes, South Korea				1.1–3.8	6.9–48.6	9.2–63.9	9.0–31.7	3.0–18.9	31.5–198.3	Yang et al. (2016)
3	Wuhan urban lakes, China			618–1014		24.6–110	22.5–48.5	40.9–50.9	30.1—51.2	68.5—145	Dou et al. (2022)
4	Twin lakes, Bhopal, India					14.1—31.2	22.1—134	16.5—72.8	19.8—301		Gupta et al. (2013)
5	Korotoa urban river, Bogra, Bangladesh				2.6–51	55–183	37–118	37–155	36–83		Islam et al. (2015)
6	Lake Geneva, Switzerland					60.3—88.9	54.7—181.4		38.8—164.7	126.8—518.2	Haller et al. (2011)
7	West Lake, Hanoi, Vietnam			329–897		26–163	24–118		30–200	101–697	Pham et al. (2007)
8	Upper Silesia region urban lakes, Poland					45.3–167.5	14.0–271.5	12.0–128.5	33.3–3125	181.7–35,200	Rzetala (2015)
9	Ashtamudi Lake, Kollam, India		6287–132,422	270–3463		180–1194	10.6–80.4	53–177	31–208	26.8–232	Hussain et al. (2020)
10	Sembakkam Lake, Chennai, India	22,575—46,500	17,213—40,745	211—609	2.27—5.10	130- 788	65—447	49—205	5.0—29	71—1009	Rosado et al. (2024a)

In contrast to heavy metal levels in water, sediment concentrations are notably higher in the urban lakes of the Pallikaranai catchment than in the Twin Lakes of Bhopal, India, except for Pb (Gupta et al., 2013). A similar situation is observed in other polluted urban lakes: West Lake, Hanoi, Vietnam (Pham et al., 2007), Wuhan urban lakes, China (Dou et al., 2022), Lake Geneva, Switzerland (Haller et al., 2011) and the Korotoa River, Bogra, Bangladesh (Islam et al., 2015). In the latter, concentrations of As are significantly higher compared to those in the urban lakes of the Pallikaranai catchment (Islam et al., 2015).

Some other polluted urban lakes exhibit remarkably high concentrations of certain heavy metals, often exceeding the levels of metals other than Pb found in the Pallikaranai catchment, while the remaining metal concentrations remain below the level in the Pallikaranai catchment. For example, urban lakes in Seoul, South Korea show much higher concentrations of Cu, while all other metals are lower (Yang et al., 2016). In the Upper Silesia region of Poland, Zn levels are significantly higher, but concentrations of all other metals are lower (Rzet al., & &a, 2015). Finally, in Ashtamudi Lake, Kollam, India, heavy metal concentrations are generally in the same range as those in the Pallikaranai catchment, with Cr and Pb being higher, Ni similar, and Cu and Zn lower (Hussain et al., 2020).

Heavy metal concentrations in the sediments of the urban lakes of the Pallikaranai catchment can be considered relatively high, except for Pb and As. Conversely, several of these water bodies exhibited lower concentrations of heavy metals in the water. This situation may suggest greater precipitation of dissolved heavy metals coming from untreated sewage in the urban lakes of the Pallikaranai catchment, potentially due to the higher pH levels observed in these lakes. Additionally, higher inputs of heavy metal-enriched particles from urban runoff that settle in the lakes, a greater proportion of atmospheric deposition, prolonged periods of contamination over time, or inherently higher background values of these metals in the soils of Chennai. It is likely that a combination of these factors is at play. Further research is needed to elucidate the specific fractions to which these metals belong, enabling a more accurate determination of their origins.

#### Comparison with sediment-quality guidelines

**Fig. 5:**
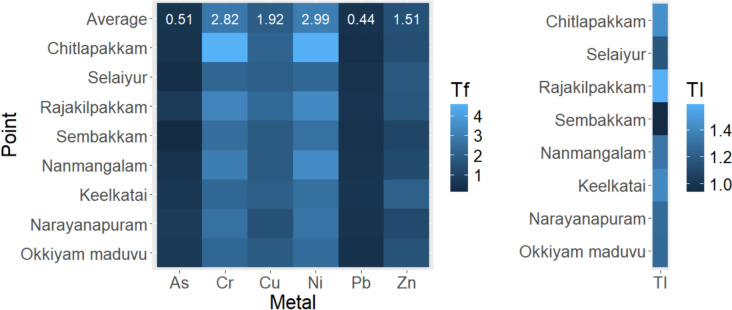
Heatmaps of the toxicity factors (Tfs) and toxicity index (TI) of heavy metals in sediments of the lakes of the Pallikaranai Catchment, Chennai, India as proposed in this article

Regarding toxicity risks, almost all the values of As and Pb fall below the TEC, indicating no toxicity risks for aquatic biota, except for As in Rajakilpakkam Lake during the second sampling (Fig. 5 and Table S7). Most Zn values are between the TEC and PEC, indicating a potential toxicity effect. However, Zn levels in Selaiyur Lake during the first sampling and Keelkatai Lake during the second sampling exceed the PEC. For Cu, concentrations are almost equally divided between those that fall between the TEC and PEC and those that exceed the PEC. Finally, all Cr and Ni concentrations are above the PEC, indicating a toxicity risk for aquatic biota from these metals.

In sediments, Cr and Ni are the metals with the highest toxicity risk. Cu and Zn are also significant contributors to toxicity risk for aquatic life. As explained for water results, a major source of heavy metals in the urban lakes of the Pallikaranai catchment is the abundant untreated sewage discharged into the lakes by nearby households. Furthermore, uncontrolled waste dumping along the lake banks exacerbates the contamination issue.

Additionally, Cr and Ni may also contribute to the sediment toxicity risk observed in the Pallikaranai catchment. Potential anthropogenic sources of Cr include electroplating, metal finishing, stainless steel production, textile dyeing, leather tanning and pigment manufacturing (Dhiman, 2026), whereas Ni is commonly associated with electroplating, alloy production and battery manufacturing (Costa et al., 2022; Peng et al., 2020). In urban environments, both metals can also enter aquatic systems through road dust, traffic-related particles, urban runoff and atmospheric deposition, including emissions from combustion processes and industrial activities (Fiala & Hwang, 2021; Gupta, 2020; Jose & Srimuruganandam, 2020; Kumar et al., 2023). Since this study measured total Cr, further Cr speciation analysis would be necessary to determine the relative contribution of Cr(III) and Cr(VI), which differ strongly in mobility, bioavailability and toxicity (Ao et al., 2025).

#### Toxicity factor and toxicity index for sediment

The average toxicity index value of metal concentrations in all sediment samples is 1.30, which falls between the TEC and PEC thresholds. In this range, some organisms may begin to experience adverse effects, though it does not necessarily imply significant harm will occur across the entire ecosystem (MacDonald et al., 2000). The metal toxicity index in sediments exhibited a slightly different trend compared to the index in water. The average of the index for two upstream lakes Selaiyur and Rajakilpakkam, was 1.38, which is higher than that of the middle lakes, Sembakkam and Nanmangalam, at 1.13. However, the index increased again in the downstream lakes, Keelkatai and Narayanapuram, reaching 1.34, before decreasing once more in Okkiyam Maduvu to 1.26. Notably, the third upstream lake, Chitlapakkam had a higher value of 1.44 compared to the 1.17 found in Selaiyur (Fig. 5).

The contrast between water and sediment results highlights the importance of assessing both compartments. Water concentrations reflected more immediate exposure conditions, with Pb and Cu contributing most strongly to water toxicity risk, whereas sediments acted as a longer-term repository of contamination, with Cr, Ni, Cu and Zn showing the highest toxicity relevance. Therefore, the combined interpretation of water- and sediment-based toxicity indices provides a more complete assessment of metal-related ecological risks than either compartment alone.

## Conclusions

This study introduced a new toxicity factor (Tf) and toxicity index (TI) for evaluating heavy metal risks in water and applied them to assess heavy metal pollution in the waters and sediments of urban lakes within the Pallikaranai catchment in Chennai, India.

The analysis of in situ parameters revealed remarkable variability within the lakes of the catchment. pH levels were neutral to alkaline, ranging between 7 and 9. Only a few measurements of electrical conductivity exceeded the common range defined by the USEPA for rivers in the United States. Dissolved oxygen levels varied greatly, with some lakes exhibiting anoxic conditions while others had concentrations exceeding 10 mg/L. This variability in parameters indicates differing impacts of untreated sewage and urban runoff on the water quality of these lakes.

Heavy metal concentrations in the water showed that Fe, Al, and Mn were the most prevalent, followed by Ni, Cu, Zn, Pb, As, and Cr. The levels of As, Cr, Ni, and Zn generally fell within Class I of the UNECE guidelines, indicating an absence of toxic effects according to this framework. However, Cu and Pb levels posed greater toxicity risks, with several samples falling into Class III and IV categories. The high input of untreated sewage and waste dumping are significant sources of Cu and Pb pollution in these lakes.

Sediment analysis revealed that the heavy metal concentrations in the Pallikaranai catchment were higher than in many other urban lakes globally, except for Pb and As. The sediment toxicity index indicated that the lakes, particularly the upstream ones, had high heavy metal concentrations, suggesting a potential risk for toxic effects. The data imply that factors such as high pH levels, atmospheric deposition, and prolonged contamination contribute to heavy metal accumulation in the sediments.

Beyond the Pallikaranai catchment, the proposed water toxicity index may be transferable to other aquatic ecosystems where heavy metal concentrations need to be translated into interpretable risk categories for monitoring and management, provided that appropriate water-quality guideline values are available.

From a policy implementation perspective, the toxicity factor and toxicity index proposed in this study can support environmental authorities by translating heavy metal concentrations in water into an easily interpretable risk score for aquatic-life protection. These tools may serve as practical screening methods to identify priority metals, rank water bodies according to potential ecological risk, and support monitoring and management decisions, particularly in urban catchments where financial and technical resources may be limited.

In the Pallikaranai catchment, the results underscore the urgent need for continuous monitoring and effective pollution mitigation strategies. Management actions should prioritize regular assessment of both water and sediments, reduction of untreated sewage inputs, control of waste dumping along lake banks, improved stormwater management, and identification of potential industrial and traffic-related metal sources.

## Supplementary Information

Below is the link to the electronic supplementary material.

Supplementary file1 (DOCX 49 KB)

## Data Availability

The datasets generated during and/or analysed during the current study are available from the corresponding author on reasonable request.
