# Supplementary material for: Water-specific toxicity factor and index for heavy metal risk assessment: application to urban lakes in Chennai, India
Source: Environ Geochem Health. 2026 Jul 8;48(10):439. doi: 10.1007/s10653-026-03298-2 (PMC13346276; doi:10.1007/s10653-026-03298-2)
Supplement: Supplementary file 1 — Supplementary file1 (DOCX 49 KB) [file 10653_2026_3298_MOESM1_ESM.docx]

# Supplementary material

Table S1. Coordinates of the sampling points.

| Point | Point | North | East |
| --- | --- | --- | --- |
| PL1 | Chitlapakkam lake | 12°55'59.4'' | 80°8'15.1'' |
| PL2 | Selaiyur lake | 12°55'0.0'' | 80°7'55.4'' |
| PL3 | Rajakilpakkam lake | 12°55'12.0'' | 80°9'2.6'' |
| PL4 | Sembakkam lake | 12°56'17.4'' | 80°9'17.5'' |
| PL5 | Nanmangalam lake | 12°56'28.7'' | 80°9'59.1'' |
| PL6 | Keelkatai lake | 12°56'59.0'' | 80°10'48.0'' |
| PL7 | Narayanapuram lake | 12°56'55.8'' | 80°12'10.3'' |
| PL8 | Okkiyam maduvu | 12°55'26.4'' | 80°13'51.2'' |

Table S2. Certified/reference values, average measured values and recovery percentages for the SO-4 certified reference material used during sediment digestion and ICP-OES analysis.

| Element | Certified/reference value | Average measured value | Recovery (%) |
| --- | --- | --- | --- |
| Al | 54,600 | 49,904 | 91.4 |
| Fe | 23,700 | 21,883 | 92.3 |
| Mn | 600 | 574 | 95.6 |
| Cr | 61 | 59.7 | 97.8 |
| Cu | 22 | 22.5 | 102.4 |
| Ni | 26 | 26.4 | 101.7 |
| Pb | 16 | 15.0 | 93.9 |
| Zn | 94 | 100.4 | 106.8 |

Table S3. In situ water quality parameters in the lakes of the Pallikaranai catchment, Chennai, India (SD: standard deviation; CV (%): coefficient of variation; Min: minimum value; Max: maximum value).

|  | Point | | Location | pH | Conductivity (µS/cm) | Dissolved oxygen (mg/L) | Oxygen saturation (%) | Temperature (^o^C) | Turbidity (NTU) |
| --- | --- | --- | --- | --- | --- | --- | --- | --- | --- |
| Sampling 1 | PL1 | | Chitlapakkam lake | 8.55 | 673 | 7.72 | 100.3 | 28.6 |  |
|  | PL2 | | Selaiyur lake | 7.20 | 1602 | 0.15 | 1.7 | 28.1 |  |
|  | PL3 | | Rajakilpakkam lake | 7.25 | 1356 | 0.43 | 5.5 | 28.3 |  |
|  | PL4 | | Sembakkam lake | 8.24 | 1435 | 7.69 | 109.4 | 29.8 |  |
|  | PL5 | | Nanmangalam lake | 7.91 | 1349 | 4.36 | 57.9 | 29.8 |  |
|  | PL6 | | Keelkatai lake | 8.82 | 1000 | 10.35 | 137.8 | 29.8 |  |
|  | PL7 | | Narayanapuram lake | 9.02 | 920 | 10.95 | 145.6 | 29.8 |  |
|  | PL8 | | Okkiyam maduvu | 7.56 | 2420 | 2.37 | 33.80 | 34.2 |  |
|  | Average | | | 8.07 | 1344 | 5.50 | 74.0 | 29.8 |  |
|  | SD | | | 0.70 | 532 | 4.28 | 57.2 | 1.9 |  |
|  | CV (%) | | | 8.69 | 39.57 | 77.83 | 77.35 | 6.46 |  |
|  | Median | | | 8.08 | 1353 | 6.03 | 79.1 | 29.8 |  |
|  | Min | | | 7.20 | 673 | 0.15 | 1.7 | 28.1 |  |
|  | Max | | | 9.02 | 2420 | 10.95 | 145.6 | 34.2 |  |
| Sampling 2. | PL1 | | Chitlapakkam lake | 8.57 | 692 | 6.89 | 95.3 | 32.7 | 19.9 |
|  | PL2 | | Selaiyur lake | 7.42 | 1274 | 0.34 | 5.0 | 31.5 | 42.2 |
|  | PL3 | | Rajakilpakkam lake | 7.11 | 1295 | 0.01 | 0.1 | 31.2 | 209 |
|  | PL4 | | Sembakkam lake | 7.62 | 1665 | 4.91 | 69.2 | 33.5 | 74.8 |
|  | PL5 | | Nanmangalam lake | 8.59 | 1185 | 8.32 | 116.2 | 33.3 | 11.1 |
|  | PL6 | | Keelkatai lake | 8.81 | 1066 | 10.64 | 150.8 | 34.0 | 25.1 |
|  | PL7 | | Narayanapuram lake | 8.91 | 873 | 10.13 | 138.8 | 31.9 | 23.9 |
|  | PL8 | | Okkiyam maduvu | 7.45 | 2250 | 0.01 | 0.1 | 33.8 | 57.1 |
|  | Average | | | 8.06 | 1288 | 5.16 | 71.9 | 32.7 | 57.9 |
|  | SD | | | 0.73 | 486 | 4.54 | 63.3 | 1.1 | 64.6 |
|  | CV (%) | | | 9.02 | 37.77 | 87.98 | 87.92 | 3.31 | 111.63 |
|  | Median | | | 8.10 | 1230 | 5.90 | 82.3 | 33.0 | 33.7 |
|  | Min | | | 7.11 | 692 | 0.01 | 0.1 | 31.2 | 11.1 |
|  | Max | | | 8.91 | 2250 | 10.64 | 150.8 | 34.0 | 209.0 |
| Average by point | PL1 | Chitlapakkam lake | | 8.56 | 683 | 7.31 | 97.8 | 30.7 |  |
|  | PL2 | Selaiyur lake | | 7.31 | 1438 | 0.25 | 3.4 | 29.8 |  |
|  | PL3 | Rajakilpakkam lake | | 7.18 | 1326 | 0.22 | 2.8 | 29.8 |  |
|  | PL4 | Sembakkam lake | | 7.93 | 1550 | 6.30 | 89.3 | 31.7 |  |
|  | PL5 | Nanmangalam lake | | 8.25 | 1267 | 6.34 | 87.1 | 31.6 |  |
|  | PL6 | Keelkatai lake | | 8.82 | 1033 | 10.50 | 144.3 | 31.9 |  |
|  | PL7 | Narayanapuram lake | | 8.97 | 897 | 10.54 | 142.2 | 30.9 |  |
|  | PL8 | Okkiyam maduvu | | 7.51 | 2335 | 1.19 | 17.0 | 34.0 |  |
| Overall | Average | | | 8.06 | 1316 | 5.33 | 72.97 | 31.3 | 57.89 |
|  | SD | | | 0.69 | 493 | 4.27 | 58.29 | 2.1 | 64.62 |
|  | CV (%) | | | 8.56 | 37.48 | 80.04 | 79.88 | 6.84 | 111.63 |
|  | Median | | | 8.08 | 1285 | 5.90 | 82.3 | 31.4 | 33.7 |
|  | Min | | | 7.11 | 673 | 0.01 | 0.1 | 28.1 | 11.1 |
|  | Max | | | 9.02 | 2420 | 10.95 | 150.8 | 34.2 | 209.0 |

Table S4. Metal concentrations (µg/L) in the waters of the urban lakes of the Pallikaranai catchment, Chennai, India (SD: standard deviation; CV (%): coefficient of variation; Min: minimum value; Max: maximum value).

|  | Point | Location | Al | Fe | Mn | As | Cr | Cu | Ni | Pb | Zn |
| --- | --- | --- | --- | --- | --- | --- | --- | --- | --- | --- | --- |
| Sampling 1 | PL1 | Chitlapakkam lake | 16.6 | 33.7 | 19.7 | 1.70 | 0.39 | 6.93 | 6.28 | 0.72 | 3.28 |
|  | PL2 | Selaiyur lake | 42.6 | 74.6 | 21.9 | 1.70 | 0.96 | 6.70 | 16.43 | 4.98 | 4.19 |
|  | PL3 | Rajakilpakkam lake | 41.8 | 48.8 | 9.1 | 0.15 | 0.44 | 6.55 | 14.48 | 6.34 | 3.19 |
|  | PL4 | Sembakkam lake | 5.8 | 4.3 | 0.4 | 1.89 | 0.32 | 7.13 | 11.74 | 2.14 | 3.15 |
|  | PL5 | Nanmangalam lake | 5.8 | 1.1 | 0.2 | 1.07 | 0.31 | 6.33 | 11.52 | 2.49 | 1.74 |
|  | PL6 | Keelkatai lake | 7.2 | 2.7 | 0.7 | 2.87 | 0.33 | 6.53 | 5.50 | 1.14 | 1.74 |
|  | PL7 | Narayanapuram lake | 13.6 | 19.8 | 8.8 | 2.10 | 0.11 | 6.82 | 4.96 | 1.42 | 3.67 |
|  | PL8 | Okkiyam maduvu | 31.8 | 67.1 | 68.9 | 2.76 | 1.53 | 1.69 | 12.62 | 3.37 | 9.33 |
|  | Average | | 20.7 | 31.5 | 16.2 | 1.78 | 0.55 | 6.09 | 10.44 | 2.83 | 3.79 |
|  | SD | | 15.8 | 29.4 | 22.9 | 0.88 | 0.47 | 1.79 | 4.34 | 1.97 | 2.40 |
|  | CV (%) | | 76.4 | 93.4 | 141.3 | 49.51 | 84.88 | 29.47 | 41.52 | 69.68 | 63.37 |
|  | Median | | 15.1 | 26.8 | 9.0 | 1.80 | 0.36 | 6.63 | 11.63 | 2.32 | 3.24 |
|  | Min | | 5.8 | 1.1 | 0.2 | 0.15 | 0.11 | 1.69 | 4.96 | 0.72 | 1.74 |
|  | Max | | 42.6 | 74.6 | 68.9 | 2.87 | 1.53 | 7.13 | 16.43 | 6.34 | 9.33 |
| Sampling 2. | PL1 | Chitlapakkam lake | 12.6 | 10.1 | 1.2 | 2.30 | 0.98 | 9.35 | 5.73 | 1.67 | 3.19 |
|  | PL2 | Selaiyur lake | 10.1 | 9.4 | 0.5 | 1.00 | 0.52 | 8.35 | 10.76 | 2.59 | 2.23 |
|  | PL3 | Rajakilpakkam lake | 8.0 | 19.8 | 6.6 | 0.93 | 0.49 | 8.79 | 10.12 | 2.22 | 6.19 |
|  | PL4 | Sembakkam lake | 7.3 | 5.5 | 0.6 | 0.41 | 0.57 | 8.47 | 6.02 | 0.85 | 3.38 |
|  | PL5 | Nanmangalam lake | 12.0 | 100.6 | 0.6 | 1.46 | 0.49 | 12.52 | 6.52 | 0.58 | 8.74 |
|  | PL6 | Keelkatai lake | 9.7 | 23.8 | 24.6 | 2.25 | 0.73 | 10.83 | 7.04 | 1.69 | 13.01 |
|  | PL7 | Narayanapuram lake | 48.9 | 98.4 | 96.7 | 1.98 | 0.49 | 8.02 | 11.18 | 0.76 | 5.13 |
|  | PL8 | Okkiyam maduvu | 15.0 | 29.3 | 0.7 | 1.66 | 0.44 | 5.20 | 4.60 | 0.70 | 5.03 |
|  | Average | | 15.5 | 37.1 | 16.4 | 1.50 | 0.59 | 8.94 | 7.75 | 1.38 | 5.86 |
|  | SD | | 13.7 | 39.3 | 33.5 | 0.68 | 0.18 | 2.14 | 2.55 | 0.77 | 3.53 |
|  | CV (%) | | 89.0 | 105.9 | 203.6 | 45.27 | 30.75 | 23.91 | 32.90 | 55.48 | 60.21 |
|  | Median | | 11.1 | 21.8 | 1.0 | 1.56 | 0.51 | 8.63 | 6.78 | 1.26 | 5.08 |
|  | Min | | 7.3 | 5.5 | 0.5 | 0.41 | 0.44 | 5.20 | 4.60 | 0.58 | 2.23 |
|  | Max | | 48.9 | 100.6 | 96.7 | 2.30 | 0.98 | 12.52 | 11.18 | 2.59 | 13.01 |
| Average by point | PL1 | Chitlapakkam lake | 14.58 | 21.88 | 10.43 | 2 | 0.69 | 8.14 | 6 | 1.2 | 3.23 |
|  | PL2 | Selaiyur lake | 26.38 | 41.98 | 11.19 | 1.35 | 0.74 | 7.53 | 13.6 | 3.79 | 3.21 |
|  | PL3 | Rajakilpakkam lake | 24.9 | 34.32 | 7.84 | 0.54 | 0.47 | 7.67 | 12.3 | 4.28 | 4.69 |
|  | PL4 | Sembakkam lake | 6.55 | 4.87 | 0.5 | 1.15 | 0.44 | 7.8 | 8.88 | 1.49 | 3.27 |
|  | PL5 | Nanmangalam lake | 8.9 | 50.83 | 0.39 | 1.27 | 0.4 | 9.43 | 9.02 | 1.54 | 5.24 |
|  | PL6 | Keelkatai lake | 8.46 | 13.25 | 12.62 | 2.56 | 0.53 | 8.68 | 6.27 | 1.42 | 7.38 |
|  | PL7 | Narayanapuram lake | 31.28 | 59.08 | 52.76 | 2.04 | 0.3 | 7.42 | 8.07 | 1.09 | 4.4 |
|  | PL8 | Okkiyam maduvu | 23.37 | 48.17 | 34.82 | 2.21 | 0.99 | 3.44 | 8.61 | 2.04 | 7.18 |
| Overall | Average | | 18.1 | 34.3 | 16.3 | 1.64 | 0.57 | 7.51 | 9.09 | 2.10 | 4.82 |
|  | SD | | 14.5 | 33.7 | 27.7 | 0.77 | 0.34 | 2.41 | 3.71 | 1.62 | 3.11 |
|  | CV (%) | | 80.6 | 98.1 | 169.7 | 47.19 | 60.13 | 32.08 | 40.76 | 77.20 | 64.39 |
|  | Median | | 12.3 | 21.8 | 3.9 | 1.70 | 0.49 | 7.03 | 8.58 | 1.68 | 3.53 |
|  | Min | | 5.8 | 1.1 | 0.2 | 0.15 | 0.11 | 1.69 | 4.60 | 0.58 | 1.74 |
|  | Max | | 48.9 | 100.6 | 96.7 | 2.87 | 1.53 | 12.52 | 16.43 | 6.34 | 13.01 |

Table S5. Toxicity factors (Tfs) and toxicity index (TI) of metals in the waters of the urban lakes of the Pallikaranai catchment, Chennai, India (SD: standard deviation; CV (%): coefficient of variation; Min: minimum value; Max: maximum value).

| Toxicity factors | | | | | | | | | | | | | | | |
| --- | --- | --- | --- | --- | --- | --- | --- | --- | --- | --- | --- | --- | --- | --- | --- |
|  | Point | | Location | | | As | Cr | | Cu | | Ni | | Pb | | Zn |
| Sampling 1 | PL1 | | Chitlapakkam lake | | | 1.17 | 1.39 | | 2.99 | | 1.42 | | 2.42 | | 1.07 |
|  | PL2 | | Selaiyur lake | | | 1.17 | 1.96 | | 2.94 | | 2.02 | | 4.02 | | 1.09 |
|  | PL3 | | Rajakilpakkam lake | | | 1.02 | 1.44 | | 2.91 | | 1.97 | | 4.04 | | 1.07 |
|  | PL4 | | Sembakkam lake | | | 1.19 | 1.32 | | 3.03 | | 1.78 | | 3.34 | | 1.07 |
|  | PL5 | | Nanmangalam lake | | | 1.11 | 1.31 | | 2.87 | | 1.77 | | 3.56 | | 1.04 |
|  | PL6 | | Keelkatai lake | | | 1.29 | 1.33 | | 2.91 | | 1.37 | | 2.69 | | 1.04 |
|  | PL7 | | Narayanapuram lake | | | 1.21 | 1.11 | | 2.96 | | 1.33 | | 2.88 | | 1.08 |
|  | PL8 | | Okkiyam maduvu | | | 1.28 | 2.11 | | 1.84 | | 1.84 | | 4.00 | | 1.21 |
|  | Average | | | | | 1.18 | 1.50 | | 2.81 | | 1.69 | | 3.37 | | 1.08 |
|  | SD | | | | | 0.09 | 0.35 | | 0.39 | | 0.28 | | 0.65 | | 0.05 |
|  | CV (%) | | | | | 7.47 | 23.13 | | 13.95 | | 16.31 | | 19.16 | | 4.92 |
|  | Median | | | | | 1.18 | 1.36 | | 2.93 | | 1.78 | | 3.45 | | 1.07 |
|  | Min | | | | | 1.02 | 1.11 | | 1.84 | | 1.33 | | 2.42 | | 1.04 |
|  | Max | | | | | 1.29 | 2.11 | | 3.03 | | 2.02 | | 4.04 | | 1.21 |
| Sampling 2. | PL1 | | Chitlapakkam lake | | | 1.23 | 1.98 | | 3.47 | | 1.38 | | 3.05 | | 1.07 |
|  | PL2 | | Selaiyur lake | | | 1.10 | 1.52 | | 3.27 | | 1.72 | | 3.62 | | 1.05 |
|  | PL3 | | Rajakilpakkam lake | | | 1.09 | 1.49 | | 3.36 | | 1.67 | | 3.39 | | 1.14 |
|  | PL4 | | Sembakkam lake | | | 1.04 | 1.57 | | 3.29 | | 1.40 | | 2.50 | | 1.08 |
|  | PL5 | | Nanmangalam lake | | | 1.15 | 1.49 | | 4.09 | | 1.43 | | 2.32 | | 1.19 |
|  | PL6 | | Keelkatai lake | | | 1.22 | 1.73 | | 3.77 | | 1.47 | | 3.06 | | 1.29 |
|  | PL7 | | Narayanapuram lake | | | 1.20 | 1.49 | | 3.20 | | 1.75 | | 2.44 | | 1.11 |
|  | PL8 | | Okkiyam maduvu | | | 1.17 | 1.44 | | 2.64 | | 1.31 | | 2.40 | | 1.11 |
|  | Average | | | | | 1.15 | 1.59 | | 3.39 | | 1.52 | | 2.85 | | 1.13 |
|  | SD | | | | | 0.07 | 0.18 | | 0.42 | | 0.17 | | 0.50 | | 0.08 |
|  | CV (%) | | | | | 5.90 | 11.42 | | 12.51 | | 11.20 | | 17.51 | | 6.94 |
|  | Median | | | | | 1.16 | 1.51 | | 3.33 | | 1.45 | | 2.77 | | 1.11 |
|  | Min | | | | | 1.04 | 1.44 | | 2.64 | | 1.31 | | 2.32 | | 1.05 |
|  | Max | | | | | 1.23 | 1.98 | | 4.09 | | 1.75 | | 3.62 | | 1.29 |
| Average by point | PL1 | | Chitlapakkam lake | | | 1.20 | 1.69 | | 3.23 | | 1.40 | | 2.73 | | 1. 07 |
|  | PL2 | | Selaiyur lake | | | 1.10 | 1.74 | | 3.11 | | 1.91 | | 4.01 | | 1.07 |
|  | PL3 | | Rajakilpakkam lake | | | 1.05 | 1.47 | | 3.14 | | 1.82 | | 4.01 | | 1.10 |
|  | PL4 | | Sembakkam lake | | | 1.12 | 1.44 | | 3.16 | | 1.59 | | 2.93 | | 1.07 |
|  | PL5 | | Nanmangalam lake | | | 1.13 | 1.40 | | 3.49 | | 1.60 | | 2.96 | | 1.12 |
|  | PL6 | | Keelkatai lake | | | 1.26 | 1.53 | | 3.34 | | 1.42 | | 2.88 | | 1.16 |
|  | PL7 | | Narayanapuram lake | | | 1.20 | 1.30 | | 3.08 | | 1.54 | | 2.66 | | 1.10 |
|  | PL8 | | Okkiyam maduvu | | | 1.22 | 1.99 | | 2.29 | | 1.57 | | 3.27 | | 1.16 |
| Overall | Average | | | | | 1.16 | 1.57 | | 3.10 | | 1.61 | | 3.18 | | 1.11 |
|  | SD | | | | | 0.08 | 0.27 | | 0.50 | | 0.24 | | 0.62 | | 0.07 |
|  | CV (%) | | | | | 6.64 | 17.58 | | 15.99 | | 14.84 | | 19.91 | | 6.24 |
|  | Median | | | | | 1.17 | 1.49 | | 3.01 | | 1.57 | | 3.05 | | 1.08 |
|  | Min | | | | | 1.02 | 1.11 | | 1.84 | | 1.31 | | 2.32 | | 1.04 |
|  | Max | | | | | 1.29 | 2.11 | | 4.09 | | 2.02 | | 4.04 | | 1.29 |
| Toxicity index | | | | | | | | | | | | | | | |
| PL1 | | PL2 | | PL3 | PL4 | | | PL5 | | PL6 | | PL7 | | PL8 | |
| 1.73 | | 1.9 | | 1.82 | 1.71 | | | 1.74 | | 1.77 | | 1.67 | | 1.73 | |

Table S6. Metal concentrations (mg/kg) in the sediments of the urban lakes of the Pallikaranai catchment, Chennai. India (SD: standard deviation; CV (%): coefficient of variation; Min: minimum value; Max: maximum value).

|  | Point | Location | Al | Fe | Mn | As | Cr | Cu | Ni | Pb | Zn |
| --- | --- | --- | --- | --- | --- | --- | --- | --- | --- | --- | --- |
| Sampling 1 | PL1 | Chitlapakkam lake | 36304 | 46912 | 949 | 5.76 | 424 | 188 | 174 | 12.9 | 294 |
|  | PL2 | Selaiyur lake | 35011 | 22675 | 165 | 4.65 | 121 | 188 | 58 | 18.4 | 626 |
|  | PL3 | Rajakilpakkam lake | 57687 | 52029 | 343 | 4.27 | 339 | 202 | 133 | 17.0 | 302 |
|  | PL4 | Sembakkam lake | 42275 | 28295 | 418 | 1.23 | 180 | 186 | 86 | 17.2 | 166 |
|  | PL5 | Nanmangalam lake | 35798 | 35746 | 859 | 3.27 | 286 | 99 | 146 | 14.0 | 148 |
|  | PL6 | Keelkatai lake | 26546 | 23668 | 1072 | 5.94 | 127 | 80 | 78 | 12.9 | 123 |
|  | PL7 | Narayanapuram lake | 29011 | 40289 | 812 | 7.90 | 152 | 87 | 71 | 16.6 | 222 |
|  | PL8 | Okkiyam maduvu | 45638 | 31735 | 339 | 9.04 | 156 | 158 | 83 | 14.6 | 349 |
|  | Average | | 38534 | 35169 | 620 | 5.26 | 223 | 149 | 104 | 15.5 | 279 |
|  | SD | | 9932 | 10665 | 340 | 2.49 | 113 | 51 | 42 | 2.1 | 162 |
|  | CV (%) | | 26 | 30 | 55 | 47.43 | 50 | 35 | 40 | 13.7 | 58 |
|  | Median | | 36051 | 33741 | 615 | 5.21 | 168 | 172 | 85 | 15.6 | 258 |
|  | Min | | 26546 | 22675 | 165 | 1.23 | 121 | 80 | 58 | 12.9 | 123 |
|  | Max | | 57687 | 52029 | 1072 | 9.04 | 424 | 202 | 174 | 18.4 | 626 |
| Sampling 2. | PL1 | Chitlapakkam lake | 32661 | 42009 | 821 | 2.61 | 371 | 159 | 172 | 10.5 | 212 |
|  | PL2 | Selaiyur lake | 44938 | 29618 | 231 | 1.20 | 147 | 111 | 67 | 14.8 | 160 |
|  | PL3 | Rajakilpakkam lake | 35076 | 25876 | 343 | 10.98 | 150 | 198 | 97 | 17.8 | 398 |
|  | PL4 | Sembakkam lake | 32001 | 24535 | 270 | 1.42 | 152 | 81 | 67 | 11.3 | 140 |
|  | PL5 | Nanmangalam lake | 34100 | 26264 | 355 | 5.68 | 158 | 160 | 92 | 20.2 | 297 |
|  | PL6 | Keelkatai lake | 28146 | 25841 | 388 | 5.38 | 153 | 218 | 72 | 26.1 | 924 |
|  | PL7 | Narayanapuram lake | 32945 | 35728 | 767 | 6.52 | 199 | 88 | 95 | 15.2 | 197 |
|  | PL8 | Okkiyam maduvu | 29434 | 22298 | 151 | 4.38 | 125 | 106 | 57 | 11.0 | 282 |
|  | Average | | 33663 | 29021 | 416 | 4.77 | 182 | 140 | 90 | 15.9 | 326 |
|  | SD | | 5098 | 6622 | 246 | 3.20 | 79 | 51 | 36 | 5.4 | 256 |
|  | CV (%) | | 15 | 23 | 59 | 67.04 | 44 | 37 | 40 | 33.9 | 78 |
|  | Median | | 32803 | 26070 | 349 | 4.88 | 153 | 135 | 82 | 15.0 | 247 |
|  | Min | | 28146 | 22298 | 151 | 1.20 | 125 | 81 | 57 | 10.5 | 140 |
|  | Max | | 44938 | 42009 | 821 | 10.98 | 371 | 218 | 172 | 26.1 | 924 |
| Average by point | PL1 | Chitlapakkam lake | 34482 | 44460 | 885 | 4.18 | 398 | 173 | 173 | 11.69 | 253 |
|  | PL2 | Selaiyur lake | 39975 | 26147 | 198 | 2.93 | 134 | 150 | 63 | 16.56 | 393 |
|  | PL3 | Rajakilpakkam lake | 46381 | 38953 | 343 | 7.62 | 244 | 200 | 115 | 17.39 | 350 |
|  | PL4 | Sembakkam lake | 37138 | 26415 | 344 | 1.33 | 166 | 133 | 76 | 14.23 | 153 |
|  | PL5 | Nanmangalam lake | 34949 | 31005 | 607 | 4.48 | 222 | 129 | 119 | 17.12 | 222 |
|  | PL6 | Keelkatai lake | 27346 | 24754 | 730 | 5.66 | 140 | 149 | 75 | 19.51 | 524 |
|  | PL7 | Narayanapuram lake | 30978 | 38008 | 789 | 7.21 | 176 | 87 | 83 | 15.9 | 209 |
|  | PL8 | Okkiyam maduvu | 37536 | 27017 | 245 | 6.71 | 141 | 132 | 70 | 12.79 | 315 |
| Overall | Average | | 36098 | 32095 | 517.6 | 5.01 | 202.5 | 144.2 | 99.65 | 15.65 | 302.4 |
|  | SD | | 8031 | 9144 | 305 | 2.78 | 96 | 50 | 38 | 4.0 | 208 |
|  | CV (%) | | 22 | 28 | 59 | 55.48 | 48 | 34 | 40 | 25.2 | 69 |
|  | Median | | 34556 | 28957 | 372 | 5.02 | 155 | 159 | 85 | 15.0 | 252 |
|  | Min | | 26546 | 22298 | 151 | 1.20 | 121 | 80 | 57 | 10.5 | 123 |
|  | Max | | 57687 | 52029 | 1072 | 10.98 | 424 | 218 | 174 | 26.1 | 924 |

Table S7. Toxicity factors and toxicity index of metals in the sediments of the urban lakes of the Pallikaranai catchment, Chennai. India (SD: standard deviation; CV (%): coefficient of variation; Min: minimum value; Max: maximum value).

| Toxicity factors | | | | | | | | | | | | | | | |
| --- | --- | --- | --- | --- | --- | --- | --- | --- | --- | --- | --- | --- | --- | --- | --- |
|  | Point | | Location | | | As | Cr | | Cu | | Ni | | Pb | | Zn |
| Sampling 1 | PL1 | | Chitlapakkam lake | | | 0.59 | 4.82 | | 2.26 | | 4.57 | | 0.36 | | 1.51 |
|  | PL2 | | Selaiyur lake | | | 0.48 | 2.09 | | 2.26 | | 2.20 | | 0.51 | | 2.36 |
|  | PL3 | | Rajakilpakkam lake | | | 0.44 | 4.06 | | 2.36 | | 3.73 | | 0.48 | | 1.53 |
|  | PL4 | | Sembakkam lake | | | 0.13 | 2.62 | | 2.25 | | 2.76 | | 0.48 | | 1.13 |
|  | PL5 | | Nanmangalam lake | | | 0.33 | 3.58 | | 1.57 | | 4.00 | | 0.39 | | 1.08 |
|  | PL6 | | Keelkatai lake | | | 0.61 | 2.15 | | 1.41 | | 2.61 | | 0.36 | | 1.01 |
|  | PL7 | | Narayanapuram lake | | | 0.81 | 2.37 | | 1.47 | | 2.46 | | 0.46 | | 1.30 |
|  | PL8 | | Okkiyam maduvu | | | 0.92 | 2.41 | | 2.06 | | 2.71 | | 0.41 | | 1.67 |
|  | Average | | | | | 0,54 | 3.01 | | 1.96 | | 3.13 | | 0.43 | | 1.45 |
|  | SD | | | | | 0.25 | 1.01 | | 0.40 | | 0.85 | | 0.06 | | 0.44 |
|  | CV (%) | | | | | 47.43 | 33.68 | | 20.49 | | 27.30 | | 13.73 | | 30.31 |
|  | Median | | | | | 0.53 | 2.51 | | 2.16 | | 2.73 | | 0.43 | | 1.41 |
|  | Min | | | | | 0.13 | 2.09 | | 1.41 | | 2.20 | | 0.36 | | 1.01 |
|  | Max | | | | | 0.92 | 4.82 | | 2.36 | | 4.57 | | 0.51 | | 2.36 |
| Sampling 2. | PL1 | | Chitlapakkam lake | | | 0.27 | 4.34 | | 2.06 | | 4.53 | | 0.29 | | 1.27 |
|  | PL2 | | Selaiyur lake | | | 0.12 | 2.33 | | 1.68 | | 2.38 | | 0.41 | | 1.12 |
|  | PL3 | | Rajakilpakkam lake | | | 1.05 | 2.35 | | 2.33 | | 3.00 | | 0.50 | | 1.82 |
|  | PL4 | | Sembakkam lake | | | 0.14 | 2.37 | | 1.42 | | 2.38 | | 0.31 | | 1.06 |
|  | PL5 | | Nanmangalam lake | | | 0.58 | 2.42 | | 2.07 | | 2.88 | | 0.57 | | 1.52 |
|  | PL6 | | Keelkatai lake | | | 0.55 | 2.38 | | 2.46 | | 2.48 | | 0.73 | | 3.01 |
|  | PL7 | | Narayanapuram lake | | | 0.67 | 2.79 | | 1.48 | | 2.95 | | 0.43 | | 1.22 |
|  | PL8 | | Okkiyam maduvu | | | 0.45 | 2.13 | | 1.63 | | 2.18 | | 0.31 | | 1.48 |
|  | Average | | | | | 0.48 | 2.64 | | 1.89 | | 2.85 | | 0.44 | | 1.56 |
|  | SD | | | | | 0.31 | 0.71 | | 0.39 | | 0.75 | | 0.15 | | 0.64 |
|  | CV (%) | | | | | 64.27 | 27.05 | | 20.82 | | 26.22 | | 33.94 | | 40.78 |
|  | Median | | | | | 0.50 | 2.37 | | 1.87 | | 2.68 | | 0.42 | | 1.37 |
|  | Min | | | | | 0.12 | 2.13 | | 1.42 | | 2.18 | | 0.29 | | 1.06 |
|  | Max | | | | | 1.05 | 4.34 | | 2.46 | | 4.53 | | 0.73 | | 3.01 |
| Average by point | PL1 | | Chitlapakkam lake | | | 0.43 | 4.58 | | 2.16 | | 4.55 | | 0.33 | | 1.39 |
|  | PL2 | | Selaiyur lake | | | 0.30 | 2.21 | | 1.97 | | 2.29 | | 0.46 | | 1.74 |
|  | PL3 | | Rajakilpakkam lake | | | 0.74 | 3.20 | | 2.34 | | 3.37 | | 0.49 | | 1.68 |
|  | PL4 | | Sembakkam lake | | | 0.14 | 2.49 | | 1.83 | | 2.57 | | 0.40 | | 1.09 |
|  | PL5 | | Nanmangalam lake | | | 0.46 | 3.00 | | 1.82 | | 3.44 | | 0.48 | | 1.30 |
|  | PL6 | | Keelkatai lake | | | 0.58 | 2.26 | | 1.94 | | 2.54 | | 0.54 | | 2.01 |
|  | PL7 | | Narayanapuram lake | | | 0.74 | 2.58 | | 1.48 | | 2.70 | | 0.44 | | 1.26 |
|  | PL8 | | Okkiyam maduvu | | | 0.69 | 2.27 | | 1.85 | | 2.44 | | 0.36 | | 1.58 |
| Overall | Average | | | | | 0.51 | 2.82 | | 1.92 | | 2.99 | | 0.44 | | 1.51 |
|  | SD | | | | | 0.27 | 0.87 | | 0.39 | | 0.79 | | 0.11 | | 0.53 |
|  | CV (%) | | | | | 54.05 | 30.75 | | 20.03 | | 26.39 | | 25.29 | | 35.31 |
|  | Median | | | | | 0.51 | 2.39 | | 2.06 | | 2.73 | | 0.42 | | 1.39 |
|  | Min | | | | | 0.12 | 2.09 | | 1.41 | | 2.18 | | 0.29 | | 1.01 |
|  | Max | | | | | 1.05 | 4.82 | | 2.46 | | 4.57 | | 0.73 | | 3.01 |
| Toxicity index | | | | | | | | | | | | | | | |
| PL1 | | PL2 | | PL3 | PL4 | | | PL5 | | PL6 | | PL7 | | PL8 | |
| 1.44 | | 1.17 | | 1.59 | 0.94 | | | 1.32 | | 1.41 | | 1.27 | | 1.26 | |
